# Digitalization in banking sector: the role of intrinsic motivation

**DOI:** 10.1016/j.heliyon.2020.e05801

**Published:** 2020-12-24

**Authors:** Ary Bastari, Anis Eliyana, Agus Syabarrudin, Zainal Arief, Alvin Permana Emur

**Affiliations:** a Universitas Negeri Jakarta, Bank Kalsel, Indonesia; b Universitas Airlangga, Indonesia; c Universitas Lambung Mangkurat, Bank Kalsel, Indonesia; d Universitas 17 Agustus 1945, PT Usaha Mulia Digital Indonesia (PT UMDI), Indonesia; e PT Usaha Mulia Digital Indonesia (PT UMDI), Indonesia

**Keywords:** Intrinsic motivation, Perceived ease of use, Perceived usefulness, Intention to use, Technology acceptance model, Quality job, Broadband access

## Abstract

The focus of this work is to test the roles of intrinsic motivation for Bank Kalsel employees regarding the use of applications and websites for task completion and performance evaluation through the TAM approach. A total of 375 staffs throughout Bank Kalsel branch offices were used as respondents in this study. Data obtained from this study were examined using the Structural Equation Modelling (SEM) by LISREL 8.8 software. The results show that intrinsic motivation, perceived ease of use, and perceived usefulness are proven to have a direct effect on the intention to use the web and applications available in the process of digitalization at Bank Kalsel. In addition, the indirect impact of the proposed model is also studied, the findings are perceived ease of use and perceived usefulness found to completely mediate the relationship.

## 1 Introduction

Along with the development of the Internet, a variety of gadgets, such as laptops, tablets, and smartphones, have been widely developed and adopted by organizations in their business operations (Dasgupta and Gupta, 2019). Various business companies that aim to survive must adapt themselves to accommodate this reality (Gobble, 2018). In fact, digitalization and adoption of technology has also drawn attention of the managers and policy makers, and it has become headlines in newspapers, magazines and practitioners' conferences (Legner et al., 2017).

New opportunities powered by digitalization are placing pressure on different companies to reconsider their existing business models and operating processes or to focus finding potential market opportunities (Bouwman et al., 2019). In Indonesia, regional banks are established to support the regional economy and are now experiencing pressure in the banking industry. This is due to the competition with private banks and national banks which are moving towards through digital banking services. One regional bank that seeks to catch up with the technology is Bank Kalsel. Since 2019 Bank Kalsel has implemented digitalization in its services and operations such as mobile banking applications, service-desk app, talentpool management app, digital performance evaluation app, and sales tracker app. In addition, digitalization at Bank Kalsel is also carried out on routine work such as digital work reports, online attendance, and online meetings.

Digitalization is considered to provide great efficiency when habits and work processes are changed to accommodate the possible efficiency improvements (Kuusisto, 2017). On the other hand, digitalization policies will surely cause changes in the normal work patterns and procedures. This process involves workers to adapt to the technology and this will have various effects on employees; some may see them as advantages and some will need time to adapt to this technology, attempting to understand and handle these changes (Saputra et al., 2020). The application of digitalization also has the potential to cause indirect cost problems that must be borne by the company and also internal resistance which will also cause concerns that lead to negative impacts (Bogodistov and Ostern, 2019; Faci et al., 2017). Then sceptical assumptions may also arise from managers and employees about the agenda of implementing digitalization which is considered to be a disruption such as time-consuming and negative impact on their productivity (El-sayed & Westrup, 2011). However, positive feelings towards the application of digitalization can reduce resistance to change, because it makes employees eager to try something new (Pipitwanichakarn and Wongtada, 2019). By looking at this matter, this research will be directed to investigate the perception of employee acceptance of digitalization conducted in Bank Kalsel through the Technology Acceptance Model (TAM).

In general, TAM is a concept which was established and empirically tested to figure out factors affecting the implementation of a technology (Davis et al., 1989; Venkatesh and Davis, 2000; Venkatesh et al., 2003). In various studies, TAM is broadly used to discover the predictors of technology acceptance, hence TAM is used in this study to analyze how the acceptance of Bank Kalsel employees towards the application of digitalization. Besides, this research investigates other factors namely Intrinsic Motivation which explores employee enjoyment, pleasure and satisfaction in the process of using application and website facilities (Chaurasia et al., 2019; Venkatesh, 2000). Next this analysis will explore the role of mediation of perceived ease of use and perceived usefulness in the proposed model, which will lead to the development of TAM-related literature.

## 2 Theoretical background

This study is directed at testing employees' acceptance on the use of applications and websites in the completion of their work assignments which is a digitalization process carried out at the Bank Kalsel. Furthermore, suggestions and reviews from previous studies are used as a theoretical basis for the formulations in this research hypothesis. To this end, this research uses TAM to examine Bank Kalsel employees’ acceptance regarding technology adoption and the digitalization process.

### 2.1 Technology acceptance model (TAM)

Originally, TAM was proposed by Davis (1986) in his doctoral thesis and has been broadly recommended as a concept to describe the predictor of technology acceptance as well as the attitudes and behaviors of end users in using information technology (website, software/application, device use) either individually or in groups (Davis et al., 1989). TAM construction was built based on behavioral concepts including Theory of Reasoned Action (TRA) (Fishbein and Ajzen, 1975), self-efficacy theory (Bandura, 1981), cost-benefit decision making procedures (Beach and Mitchell, 1978), Theory Diffusion of Innovation (IDT) (Tornatzky and Klein, 1982), Channel Disposition Model (Swanson, 1987). Furthermore, Davis is of the view that user acceptance upon technology is shown through two main TAM elements namely Perceived ease of use and Perceived Usefulness.

TAM has been gradually developed into other version, TAM 2 (Venkatesh and Davis, 2000), TAM 3 (Venkatesh and Bala, 2008), and the latest version called *Universal Theory of Acceptance and Use of Technology* (UTAUT) (Venkatesh et al., 2003). Venkatesh et al. (2012) argued that UTAUT was first designed to describe the acceptance and use of technology by employees, then a trial was conducted to examine the construction of UTAUT in other broader context that is consumer technology. Until now UTAUT has been applied to various technology adoption studies and is considered to have internal reliability in various studies (Kemp et al., 2019). However, there is an assumption that the utility of UTAUT is not universal and tends to be rigid (Ros et al., 2015), this is different from the more flexible TAM structure (Kemp et al., 2019; Ros et al., 2015).

External variables can be found in a study of TAM construction in the form of system design, practice, records and further forms of support of policymakers who can affect the use of the technology (Davis, 1989). This research is specifically directed to look at the intrinsic motivation of Bank Kalsel employees in using applications/websites that are part of the digitalization process at Bank Kalsel. Intrinsic motivation or referred to as perceived enjoyment is considered as a strong construct related to the core concept of TAM (i.e., perceived usefulness and perceived ease of use) to explain attitudes in technology use (Alalwan et al., 2018; Rouibah et al., 2016; Shiau and Luo, 2013; Teo and Noyes, 2011). Furthermore, the conceptual framework of this study borrows constructions from theory and models from previous research by adding and modifying certain items to be applicable to the context of digitalization in Bank Kalsel. This research proposes intrinsic motivation as the external variable of TAM construction, namely perceived ease of use, perceived usefulness and the next variable is intention to use. It is specifically necessary to observe the behavior of Bank Kalsel employees and determine the intensity of finding company performance appraisal information, announcements, and latest reports. In addition, this is intended to adjust to the working conditions and evaluation needs related to the digitalization policy in the Bank Kalsel.

### 2.2 intrinsic motivation

Motivation has a role in formulating a person's behavior and actions (Zhang et al., 2008). When someone is not motivated, that person will not feel the urge or inspiration to act on something. Motivation can be driven intrinsically and extrinsically, which are both tailored to diverse reasons or goals for taking an action (Lee et al., 2006; Teo and Noyes, 2011; Wang et al., 2012). Intrinsic motivation is linked to activities carried out for individual satisfaction that is separated from the consequences, benefits, and objectives of the activity (Ryan and Deci, 2000). Intrinsic motivation has also been debated as a major determinant of participation in any activity (Hsu, 2017). In addition, intrinsic motivation can be expressed as the perception of doing an activity by users for pleasure and satisfaction, and intrinsically motivated behavior will be voluntary and self-determined and optimally engage individuals in certain activities that they find interesting, new, and challenging (Chaurasia et al., 2019). Therefore, intrinsic motivation can be inferred from an appreciation of activity. Intrinsic motivation which is commonly interpreted as perceived enjoyment refers to the extent to which technological activities is considered pleasant regardless of the job consequences (Davis et al., 1992). It can be concluded that intrinsic motivation or perceived enjoyment, in this context, is mainly related to the process of carrying out the activity itself.

Furthermore, there are two general attitudes of intrinsic motivation that are represented as perceived enjoyment (Sun, 2008). First the perceived enjoyment is not stable, but rather can continually change. Therefore, users can see a web system/app being fun at one time but maybe not at another time. Second, perceived enjoyment has no situational consistency and depends on the web/app system. This shows that basically the perceived enjoyment measures how much fun the individuals feel their interaction with the web/app system.

Perceived enjoyment in the use of a technology is considered a central component of many current affective principles (Sun, 2008). For some studies, perceived enjoyment is an essential factor of many other affective theories such as flow experience (Hsu and Lu, 2004; Webster and Martocchio, 1995; Zhou, 2012, 2013), perceived playfulness (Lee et al., 2006; Padilla -Meléndez et al., 2013), and cognitive absorption (Saadé and Bahli, 2005; Zhang, Li and Sun, 2006). Furthermore, perceived enjoyment is commonly used by researchers who researched human reactions to technological acceptance (Alalwan et al., 2018; Dickinger et al., 2008; Koenig-Lewis et al., 2015; Shiau and Luo, 2013; Venkatesh, 2013). , 2000). By considering this construct and trailing the definition of Davis et al. (1992), the analysis will consistently choose Intrinsic Motivation to illustrate that this is an impulse that originates from a person.

### 2.3 Perceived ease of use

It is a point where someone feels that using IT means free of work or other activities (Davis, 1989). To make it simple, Dickinger et al. (2008) defined perceived ease of use as a visible and understandable perception of use within a system. In practice, it is defined as an understanding of an individual not having to make an effort to operate certain IS technologies (Venkatesh, 2000). Thus, this easiness is also linked with giving easier operating opportunities for employees (Donmez-Turan, 2019) Perceived ease of use in this context is the perception and employee trust level in technology adoption and digitalization in the workplace through various available applications and websites that will facilitate the work they do.

### 2.4 Perceived usefulness

It is someone's confidence that using certain information technology system will in turn improve the performance of the work (Davis, 1989; Venkatesh, 2000). In addition, perceived usefulness discusses technology acceptance related to extrinsic functions and benefits in using technology (Venkatesh and Bala, 2008). Thus, perceived usefulness also acts as extrinsic motivation in an activity using information technology systems (Alalwan et al., 2018; Lee et al., 2015; Zhang et al., 2008). This is because perceived usefulness has a role in achieving valuable results that differ from the activities of the use of information technology systems, such as performance improvement, salary, or job promotion (Alalwan et al., 2018). These benefits can be attributed to the extent to which employees consider using applications and websites in the digitalization process at the Bank Kalsel as a more efficient way of completing tasks, saving more time and energy in using applications and websites compared to using manual methods to do work with specific goals same.

### 2.5 Intention to use

In numerous TAM studies, intention to use is defined as a fundamental prerequisite for the actual actions of the person when implementing systems and using new technologies (Alalwan et al., 2018; Marakarkandy et al., 2017). Besides, intention to use is also described as an interest to continually participate or take part in a particular system (Islam and Mäntymäki, 2011; Venkatesh and Bala, 2008). According to Law (2020), intention to use is also related to the user's intention to continue using the system or technology after initial use. Referring to the technology acceptance model (TAM), that intention to use is a key factor in determining the actual use of technology systems in the future. Intention to use as a behavioral construct can be accepted as a representation of attitude, behavior and actual use. This may occur because intention to use is a reflection of knowledge regarding technology adoption that leads to a person's behavior. This is supported by a statement regarding the technology acceptance model, that intention to use will determine use behavior (Purwanto and Loisa, 2020). Individual intention is known to determine user behavior. Behavior is defined as the intention to use someone in carrying out certain actions (Gupta and Arora, 2019). Therefore, intention to use can predict the appropriate behavior as long as the person takes action voluntarily (Purwanto and Loisa, 2020). In the technology acceptance model, actual usage behavior is modeled as a direct function of behavioral intention (Hossain et al., 2017). In addition, in the construction of the technology acceptance model developed by Davis (1989), a person's intention to use technology is also determined by attitude. Previous studies have found the effect of attitude on the intention to use of an application in the context of self-service banking technology (Fogarty and Rose, 2006). Furthermore, in this study, employees did not make the decision in adopting applications that became a means of digitalization in Bank Kalsel, because this activity is determined by the company (Hernández et al., 2008). The intention to use in this context is more directed to see the employees' intensity in seeing and observing the results of their performance appraisal, reports on work progress, and updates on the development of daily information available in the application and website of the Bank Kalsel. Obviously, each employee might have different intentions in accessing applications and websites that show changes in performance value and incentives obtained, so investigating this as a form of digitalization acceptance will show how enthusiastic the employees are and how important this is to them.

### 2.6 Research model & hypothesis

In TAM construction, Davis et al. (1992) proposed that perceived enjoyment is synonymous with intrinsic motivation affecting an activity and it is not linked to other purpose than the activity process itself. In addition, Venkatesh and Speier (2000) compared conventional training methods with game-based training, the results showed that game-based training methods designed to enhance intrinsic motivation yielded greater perceived enjoyment and perceived ease of use than conventional training methods. Then, Venkatesh (2000) discovered that the effect of perceived enjoyment on perceived ease of use is greater because the users encounter the system more directly over time. This result also indicates that perceived ease of use is affected by how fun is using the program. Moreover, Davis et al. (1992) noted that usefulness refers to extrinsic motivation and enjoyment refers to intrinsic motivation and both are substantial determinants of Intention to use.

Various studies have described a major effect of perceived enjoyment on perceived ease of use, perceived usefulness, and intention to use (Alalwan et al., 2018; Koenig-Lewis et al., 2015; Rouibah et al., 2016; Shiau and Luo, 2013; Zhang et al., 2008). Alalwan et al. (2018) tested the acceptance of mobile internet to one provider in Saudi Arabia and found that perceived enjoyment served as an intrinsic utility of users and an important predictor in determining customer intentions and perceived usefulness. In research by Salloum et al. (2019), perceived enjoyment is something that captures distinct characteristics of users and is considered to have a positive impact on perceived easiness and perceived usefulness. It means, perceived enjoyment as intrinsic motivation has been confirmed as a good predictor to determine variables in TAM approach. In addition, the placement of perceived enjoyment as intrinsic motivation on external variables TAM can be considered as robust. Furthermore, hypotheses in this study are proposed as follows:

H1 Intrinsic motivation of Bank Kalsel employees in using applications and web has a positive effect on the intention to use applications and web as part of the digitalization process in Bank Kalsel.

H2 Intrinsic motivation of Bank Kalsel employees in using applications and web has a positive effect on the perceived ease of use applications and web as part of the digitalization process in Bank Kalsel.

H3 Intrinsic motivation of Bank Kalsel employees in using applications and web has a positive effect on the perceived usefulness of applications and web as part of the digitalization process in Bank Kalsel.

Perceived ease of use is connected with basic types of usage characteristics (Dickinger et al., 2008). In this case, Bank Kalsel's digitalization process through applications and websites may be viewed as emerging technology that will allow employees to have a certain degree of expertise and awareness to use in a secure and productive manner. In the initial literature, TAM (Davis et al., 1989; Venkatesh and Davis, 2000), agreed on the effect of perceived ease of use on intention to use.

Recent research focusing on technology adoption often support the connection between perceived ease of use and intention to use (Alalwan et al., 2018; Bashir and Madhavaiah, 2015; Koenig-Lewis et al., 2015; Rouibah et al., 2016; Saadé and Bahli, 2005; Zhang et al., 2008). In other contexts, such as e-learning systems, the notable influence of perceived ease of use on intention to use has been well tested (Salloum et al., 2019; S. Zhang et al., 2008), Service mobile apps (Leon, 2018), Mobile commerce, and Tourism mobile app (Chen and Tsai, 2017). However, the relationship between the two is often found to be insignificant, for example in the Internet banking context in India (Chen and Tsai, 2017) and the Text Mining System (TMS) in the US and Europe (Demoulin and Coussement, 2020). This might be due to differences in user preferences as well as cultural differences in developed and developing countries related to technology (Salloum et al., 2019). Taking this into consideration, this study will further examine this relationship in the context of the digitalization process at the Bank Kalsel. The hypothesis is proposed as follows:

H4 Perceived ease of use of Bank Kalsel employees in using applications and websites has a positive effect on intention to use applications and web as part of the digitalization process at Bank Kalsel.

Perceived usefulness is the degree to which individuals find technology as a more efficient means of doing tasks, in terms of time and energy compared to conventional means to access the same form of service (Davis, 1989; Venkatesh and Bala, 2008; Venkatesh and Davis, 2000). Digitalization often provides higher mobility that allows users to use technology available anytime and anywhere without restrictions. Thus, this makes employees more motivated to consider using applications and the web as something useful in their daily lives (Alalwan et al., 2018; Pousttchi and Goeke, 2011).

Many TAM studies have found the role of perceived usefulness on intention to use (Fan et al., 2013; Langer et al., 2018; Ros et al., 2015). A study by Koenig-Lewis et al. (2015) regarding the adoption of Mobile Payment by young people in France confirmed this relationship. Then research by Hernández et al. (2008) regarding the application of decision-making technology in business organizations shows perceived usefulness as a good predictor in determining the intensity of use. If users consider that technology can provide additional benefits and productivity, they will be interested to operate the system. As a result, perceived usefulness may affect intention to use. However, several studies also found this relationship was not significant, such as a research in Nepal by Teo et al. (2019), where the results show the unsupported impact of perceived usefulness on intention to use in the context of internet learning. The same finding was also shown in Bashir and Madhavaiah (2015) study which examined the acceptance of Internet Banking in India. This difference in results shows that the technological characteristics, user preferences, and culture influence these results. Furthermore, this study will examine this relationship in the context of using applications and websites as part of the digitalization process at the Bank Kalsel. Thus, the following is the proposed hypothesis:

H5 Perceived usefulness of Bank Kalsel employees in using applications and websites has a positive effect on intention to use applications and web as part of the digitalization process in Bank Kalsel.

According to the early research of TAM (Davis, 1989), the impact of TAM's external variables on the intention to use is affected by perceived ease of use and perceived usefulness. As TAM is more broadly adopted in various information technology contexts, perceived ease of use and perceived usefulness are also proven to have a mediating effect on the influence of external variables on intention to use (Balog, 2015; Jackson et al., 2013; Ramayah, 2003; Sánchez & Hueros, 2010; Venkatesh, 2000). In Moslehpour's research (2018), which discusses consumer acceptance of the adoption of e-purchase applications in Taiwan, found the notable role of perceived ease of use and perceived usefulness as a full mediator of individual determinants namely conscientiousness towards intention to use. Then in the study regarding E-Learning at two Romanian universities, Balog (2015) in his research found mediation of perceived ease of use and perceived usefulness in the effect of perceived enjoyment on behavioral intention.

In testing the role of perceived ease of use and perceived usefulness some studies do not find supportive results either. For example, research conducted by Guriting and Ndubisi (2002) did not find an indirect effect between individual utilities namely computer experience and behavioral intention through perceived ease of use and perceived usefulness in online banking application in Malaysia. Then unsupported perceived ease of use and perceived usefulness as mediators of the external relations of variables on attitude and intention to use are also found in the study by Lu et al. (2009) who tested the acceptance of self-check-in service at airlines in Taiwan. Because of these different findings, this study proposes to test it in the context of using applications and websites as part of digitalization at Bank Kalsel. The research hypothesis is as follows:

H6 Perceived ease of use of Bank Kalsel employees in using applications and websites mediates their intrinsic motivation towards the intention to use applications and web as part of the digitalization process at Bank Kalsel.

H7 Perceived usefulness of Bank Kalsel employees in using applications and websites mediates their intrinsic motivation towards the intention to use applications and the web as part of the digitalization process at Bank Kalsel.

**Figure 1:**
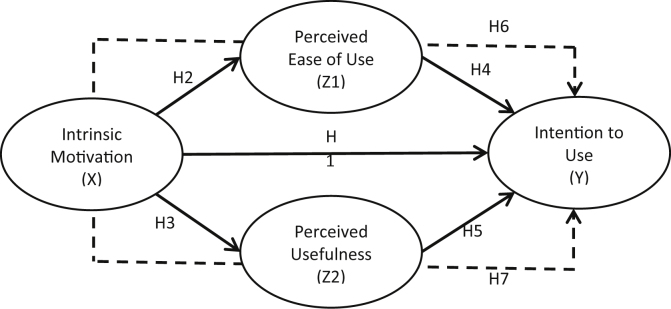
Conceptual framework.

The aforementioned Hypotheses are illustrated in Figure 1 below:

## 3 Research design and method

### 3.1 Methods

This research is known to use a quantitative approach through a survey of all employees in 91 branch offices of Bank Kalsel in Indonesia. After the required data is collected, the next stage is carried out, namely the analysis technique. In this study, the data that has been obtained will be tested using LISREL 8.8 software as part of Structural Equation Modeling (SEM). LISREL is a statistical software that will be used in structural equation modeling in producing the results of statistical analysis and calculation factor analysis. LISREL software itself has advantages, such as having the ability to identify relationships between complex variables. However, LISREL also has drawbacks such as its inability to process SEM data with a small sample size (Hair et al., 2010).

The method in research is in accordance with ethical service procedures. In Indonesia, the banking system has become part of a conceptual framework related to regulations set by the Financial Services Authority (OJK). Therefore, the implementation of the digitalization process has its own limitations as stipulated by the OJK. Bank Kalsel itself in the digitalization process uses applications and websites, which have met the legal instruments of the OJK and Bank Indonesia. In developing bank-side needs, the digitalization process using applications and websites is an important tool. Furthermore, the policy of the President Director ethically recognizes this research to support further behavior in business processes, through digitalization using applications and websites.

This study has also received ethical approval by Universitas Airlangga, specifically by its research and development division. Research and Innovation Institutes of Universitas Airlangga which is now known as Development and Innovation Institute for Publishing Journal and Intellectual Property Rights (LIPJIPHKI) is an institute in Universitas Airlangga which is responsible to supervise several areas, namely Publications and Journals, Innovation and Intellectual Property Rights, as well as Publishing/Airlangga University Press. Moreover, it is in charge of developing research and directing the results of innovative research products for the benefit of the community. This Institute also has the authority to give ethical approval for research done by lecturers’ of Universitas Airlangga or taken place in Universitas Airlangga.

### 3.2 Data collection procedure

Data in the research model was collected from Bank Kalsel staff employees at 91 Bank Kalsel branch offices in Indonesia. The survey was conducted by distributing Google Form questionnaires to all employees and the results were collected data of 556 respondents. Hence, it is known that the population in the study was 556 respondents who came from 91 branch offices of Bank Kalsel in Indonesia. Then the data is filtered to take staffs that are permanent and have worked for more than two years. The sampling technique is thus called purposive sampling which ultimately collected a sample of 375 respondents which will then be tested in this study. Those respondents were also selected because their work interacts more with technology systems. Furthermore, the description of respondents is explained in the following table:

As displayed in Table 1, the number of male respondents (206 employees) exceeds the number of female respondents. Based on the last education, 316 respondents or equal to 84,3% of all the respondents hold Bachelor degree as the last educational background followed by Master degree (10,1%), Diploma (3,7%) and High School (1,9%). For the marital status, the majority of the respondents (83,2%) stated that they are married, while the 14,7% are single and 2,1% are divorced. Furthermore, regarding the years of working, most of the respondents or as many as 181 employees have been working for more than 8 years in Bank Kalsel.

**Table 1:** Descriptive statistics.

	%	Frequency
**Gender**		
Male	54,9	206
Female	45,1	169
**Education**		
High School	1,9	7
Diploma	3,7	14
Bachelor	84,3	316
Master	10,1	38
**Marital Status**		
Single	14,7	55
Married	83,2	312
Divorce	2,1	8
**Length of Work**		
2 Years	12,0	45
3–4 Years	12,0	45
5–6 Years	15,5	58
7–8 Years	12,3	46
>8 Years	48,3	181
**Age**		
20–25	2,7	101
26–30	35,5	133
31–35	36,3	136
36–40	16,3	61
41–45	6,1	23
46–50	2,1	8
51–55	1,1	4

### 3.3 Measure

In the questionnaire, 9 items that were used to measure intrinsic motivation were taken from Venkatesh (2000), Dickinger et al. (2008), and Huang et al. (2019) as an example “I enjoyed experiencing digital applications of the Bank Kalsel (web/android) very much.”. Then 8 items perceived ease of use and 9 items perceived usefulness in this study were taken from previous research namely Hansen et al. (2018) & Leon (2018) one example of “I feel confident understanding terms/words relating to computer software to support my job with digital applications of Bank Kalsel (web/android)” & “Digital applications of Bank Kalsel (web/android) increases my productivity”. Furthermore 12 items to measure Intenton to use were also taken from previous TAM research Ismail (2016) & Leon (2018) such as “I plan to use digital applications of Bank Kalsel (web/android) in my daily life”. All items used in this study were translated, validated and modified to suit the specific technology studied in this study. Then the answers from 38 items using a Likert scale (1–5) questionnaire, ranging from “strongly disagree”' to “strongly agree”.

### 3.4 Data analysis

The tests on research models use a two-stage structural equation model with confirmatory factor analysis (CFA) and structural equation models (SEM). This study, thus, applies structural equation modelling with a robust likelihood estimation method, because the data of this study do not meet the normality (Bentler, 2006; Chou et al., 1991; Hu et al., 1992). Furthermore, data testing is done by confirmatory factor analysis (CFA) to evaluate the construct validity of convergent, discriminate validity. Then, the structural equation model (SEM) is tested for the nomological validity and the effect of the model latent construct. Furthermore, this study also tested the indirect effect or mediating effect with four stages of the conditions proposed by Baron and Kenny (1986), after which Sobel Test was done to find out the strength of the indirect effect proposed in this study.

## 4 Results

### 4.1 Measurement model

Reliability testing that has been done shows that the reliability coefficient of intrinsic motivation is 0.95, perceived ease of use is 0.94, perceived usefulness is 0.94, and intention to use is 0.95. This shows the composite reliability of all variables all exceeding the threshold value of 0.70 which establishes the reliability of the construction in this study. Furthermore, to test the validity of the variables of this study seen from the results of the convergent validity assessed through the Average Variance Extracted (AVE) test. The results show AVE intrinsic motivation value of 0.63, perceived ease of use of 0.56, perceived usefulness of 0.63, and intention to use of 0.57. These results support the convergent validity of all variables because AVE exceeds the standard threshold value of 0.50. The overall results of CFA testing are shown below:

Table 2 shows that all variables have met the research requirements, namely the validity and reliability requirements. This can be seen through the intrinsic motivation variable, perceived ease of use, perceived usefulness, intention to use which shows that the SFL (standard loading factor) results are ≥0.5, the CR (composite reliability) results are ≥0.7, and the AVE (average variance extracted results) has been ≥0.5.

**Table 2:** CFA result.

Variable/Indicator	SFL ≥0.5	Error	CR ≥ 0.7	VE ≥ 0.5
**Intrinsic Motivation**			0,95	0,63
IM1	0,87	0,24		
IM2	0,89	0,22		
IM3	0,8	0,37		
IM4	0,88	0,22		
IM5	0,59	0,65		
IM6	0,9	0,20		
IM7	0,88	0,23		
IM8	0,8	0,36		
IM9	0,84	0,29		
**Perceived Ease of Use**			0,94	0,56
PEU1	0,67	0,54		
PEU2	0,7	0,51		
PEU3	0,68	0,54		
PEU4	0,77	0,40		
PEU5	0,75	0,44		
PEU6	0,81	0,34		
PEU7	0,84	0,30		
PEU8	0,81	0,34		
**Perceived Usefulness**			0,94	0,63
PU1	0,8	0,36		
PU2	0,84	0,29		
PU3	0,88	0,23		
PU4	0,86	0,25		
PU5	0,85	0,27		
PU6	0,8	0,37		
PU7	0,76	0,42		
PU8	0,73	0,47		
PU9	0,66	0,56		
**Intention of Use**			0,95	0,57
IU1	0,75	0,44		
IU2	0,82	0,34		
IU3	0,81	0,34		
IU4	0,82	0,33		
IU5	0,79	0,38		
IU6	0,79	0,38		
IU7	0,83	0,31		
IU8	0,81	0,34		
IU9	0,74	0,46		
IU10	0,71	0,50		
IU11	0,65	0,59		
IU12	0,78	0,39		

Furthermore, goodness of fit is tested using various indexes of absolute, incremental, and parsimony fit. The results in Table 3 below show various tests of goodness of fit (χ2 = 3215.08, df = 660, p ≤ .0000), Normed Fit Index (NFI = 0.96), Non-Normed Fit Index (NNFI = 0.97), Comparative Fit Index (CFI = 0.97), Incremental Fit Index (IFI = 0.97), Relative Fit Index (RFI = 0.96), Standardized Root Mean Square Residual (SRMR = 0.028) have values above the cut of value. Then at pχ2 (p-value = 0,000) the criteria of Root Mean Square Error of Approximation (RMSEA = 0.10) Goodness-of-Fit Index (GFI = 0.69), Adjusted Goodness of Fit Index (AGFI = 0.65) shows weakness in the model. This is still quite appropriate according to Hu and Bentler (1999) and, where the model can be stated to have a goodness of fit that is quite appropriate.

**Table 3:**
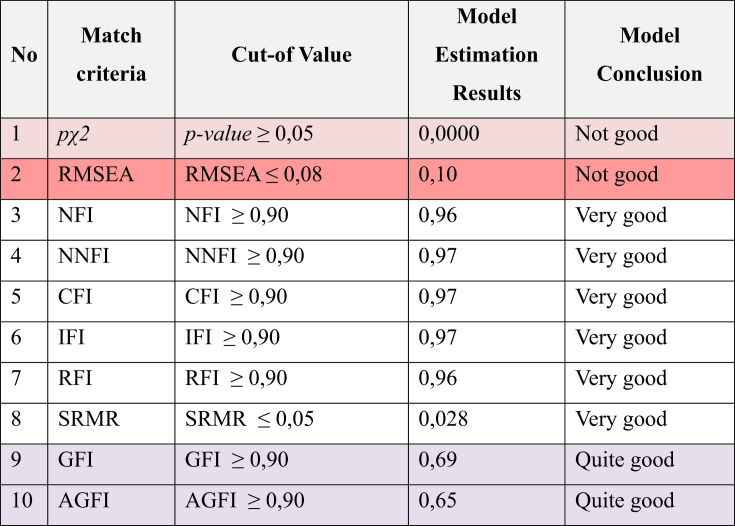
Goodness of fit results.

### 4.2 Structural model testing

**Figure 2:**
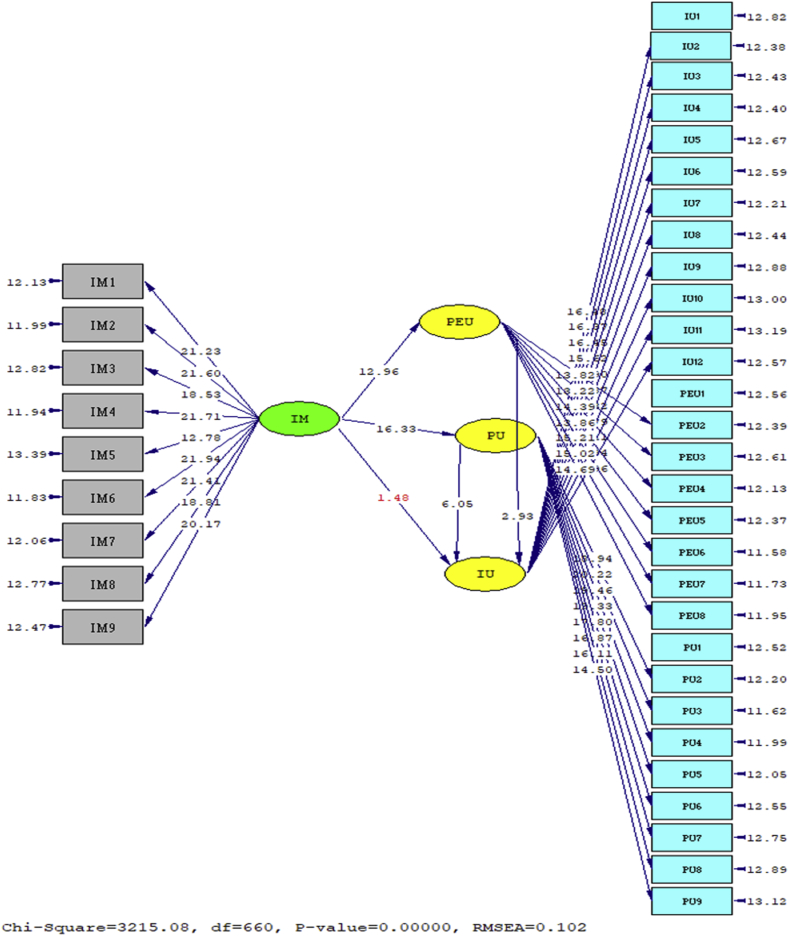
t-Value diagram.

Structural model test results show that the intrinsic motivation of Bank Kalsel employees positively and significantly influence perceived ease of use (t = 12.96 > 1.64) and perceived usefulness (t = 16.33 > 1.64) employees in using website and application in the digitalization process at the Bank Kalsel. These results indicate the second hypothesis and the third hypothesis are statistically supported. Then intrinsic motivation of Bank Kalsel employees in the structural testing model was not proven to affect intention to use (t = 1.48 > 1.64) websites and applications in the digitalization process at Bank Kalsel, this at the same time rejected the first hypothesis of this study. Furthermore, the structural testing model also showed positive effect of perceived ease of use on intention to use (t = 2.93 > 1.64) and perceived usefulness on intention to use (t = 6.05 > 1.64). Thus, these results indicate the greater perceived ease of use and perceived usefulness that comes from the employees of the Bank Kalsel will increase their intention to use in accessing websites and applications in the process of digitalization at the Bank Kalsel, so that the fourth hypothesis and the fifth hypothesis proposed by this study are supported. The results of t-statistics in the research model are displayed in Figure 2 below and Table 4.

**Table 4:** Result of direct effect test.

Path	t-count	t-table (critical value)	Path Coefficient		R-square	Conclusion Hypothesis
			Direct	Total Effect		
IM → IU	**1,48**	1,64	0,15	0,15	0,66	**Rejected**
IM→ PEU	12,96	1,64	0,79	0,79	0,62	Accepted
IM → PU	16,33	1,64	0,88	0,88	0,78	Accepted
PEU → IU	2,93	1,64	0,18	0,18	0,66	Accepted
PU → IU	6,05	1,64	0,54	0,54	0,66	Accepted

Related to the value of the path produced, intrinsic motivation towards intention to use shows a path value of 0.15. Then the value of the path produced by intrinsic motivation towards perceived ease of use is 0.79, the result is lower than the value of the path intrinsic motivation towards perceived usefulness that is equal to 0.88. Furthermore, the path value generated by perceived ease of use of intention to use is 0.18, this result is also lower when compared to the path value of perceived usefulness of intention to use which has a value of 0.54.

**Figure 3:**
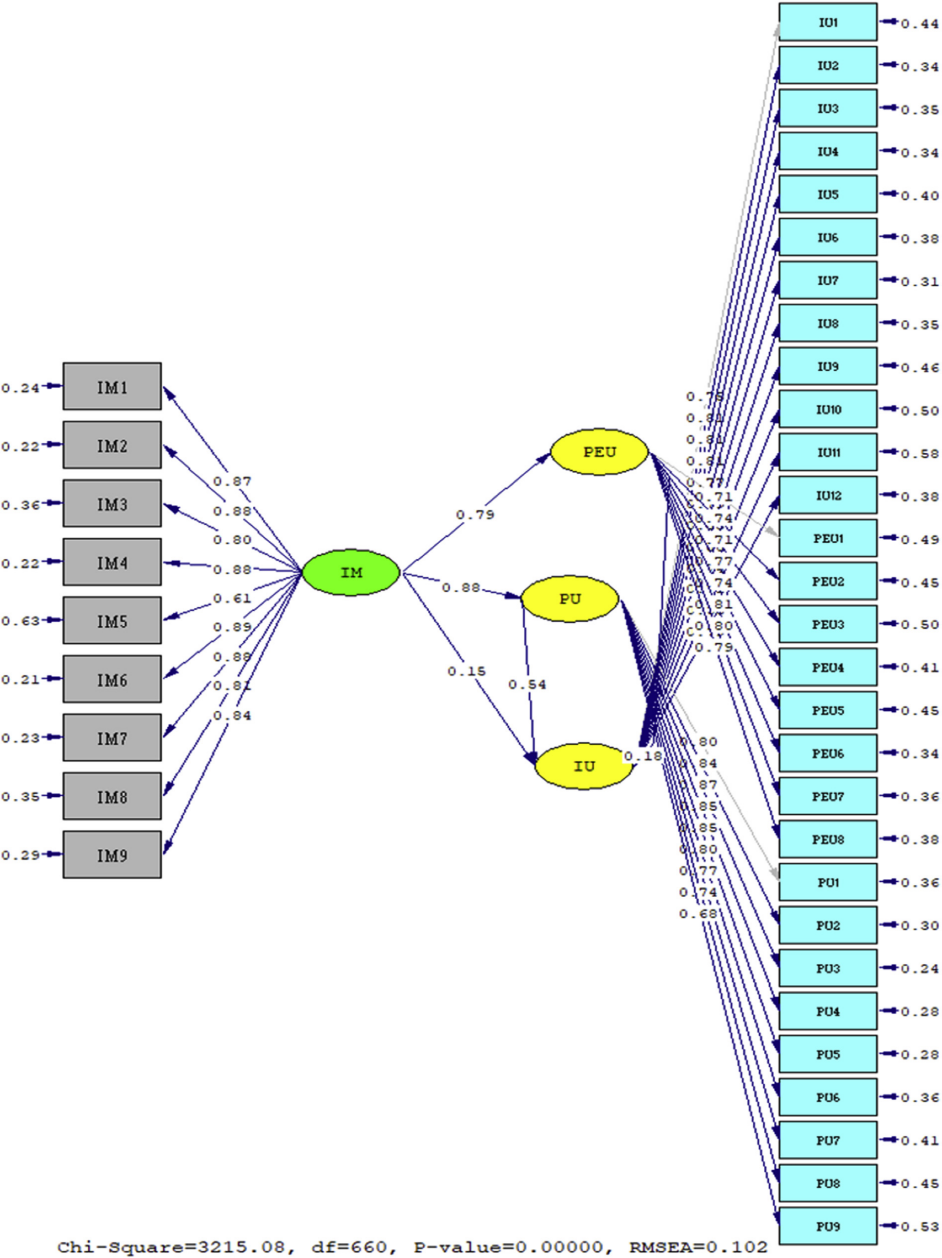
Path diagram.

The results of path coefficient test are illustrated in Figure 3 and Table 4.

### 4.3 Indirect effect test

Sobel Test is used to see the indirect effects in the proposed model. The results confirm that the impact of intrinsic motivation on intention to use through perceived ease of use are statistically significant (t = 2.84 > 1.64), this also supports the sixth hypothesis which states that employees’ perceived ease of use mediates their intrinsic motivation towards the intention to use applications and the web that are part of the digitalization process at the Bank Kalsel. Then the Sobel Test analysis also shows the significance of the effect of intrinsic motivation on intention to use through perceived usefulness (t = 5.66 > 1.64), the results also support the seventh hypothesis which states the perceived usefulness of Bank Kalsel employees in using applications and the website mediates their intrinsic motivation towards the intention to use applications and the web that are part of the digitalization process at the Bank Kalsel. Thus, the overall mediation relationship in this study is full mediation in which the variables that act as mediators and independent variables are jointly tested for their influence on the dependent variable, and the result is the independent variable in this research model that is intrinsic motivation becomes insignificant (Baron M. & Kenny A., 1986).

## 5 Discussion

The results of this research test show a significant impact of intrinsic motivation on perceived ease of use and perceived usefulness. This is shown in Table 4 which produces the Result of Direct Effect Test analysis. The effect of intrinsic motivation on perceived ease of use has a t-value of 12.96, which means > 1.64. The influence of intrinsic motivation on perceived usefulness has a t-value of 16.33, which also means > 1.64. Hence, it can be stated that when the intrinsic motivation felt by employees is getting higher, then the perceived ease of use and perceived usefulness of employees will also increase. This finding supports previous studies regarding the acceptance of technology use (Alalwan et al., 2018; Koenig-Lewis et al., 2015; Padilla-Meléndez et al., 2013; Teo and Noyes, 2011).

These results show that the high enjoyment felt by the employees of Bank Kalsel will affect their high perception of the ease and benefits obtained by digitalization the Bank Kalsel through various applications and websites. This also explains that with digitalization, Bank Kalsel employees will gain more experience working directly with the system.

This study discovered that the effect of intrinsic motivation on intention to use was not significant. This is shown in Table 4 which produces the Result of Direct Effect Test analysis. The influence of intrinsic motivation on intention to use has a t-value of 1.48, which means < 1.64. These findings provide support in previous studies regarding the acceptance of technology that uses perceived enjoyment as an external variable at TAM (Balog, 2015; Koenig-Lewis et al., 2015; Sun, 2008). This shows intrinsic motivation which is the enjoyment felt by Bank Kalsel employees in using available applications and the web is not an important factor for them to access applications and the web.

Furthermore, the testing of this study shows a significant effect on perceived ease of use and perceived usefulness on intention to use. This is shown in Table 4 which displays the Result of Direct Effect Test analysis. The effect of perceived ease of use on intention to use has a t-count value of 2.93, which means > 1.64. Meanwhile, the effect of perceived usefulness on intention to use has a t-value of 6.05, which means it is also> 1.64. These findings provide support for various recent studies in the scope of technology acceptance related to the relationship between perceived ease of use and intention to use (Alalwan et al., 2018; Bashir and Madhavaiah, 2015; Koenig-Lewis et al., 2015; Rouibah et al., 2016; Saadé and Bahli, 2005; Zhang et al., 2008). This finding is also consistent with many TAM studies that have found a significant effect of perceived usefulness on intention to use (Fan et al., 2013; Langer et al., 2018; Ros et al., 2015). This shows that the employees of Bank Kalsel already have a certain level of experience and knowledge to use the web and applications safely and efficiently. Because through perceived ease of use, employees will use new applications and the web without certain difficulties because they believe that the system is easy to learn or use. In addition, they are also supported by perceived usefulness, which makes Bank Kalsel employees consider using applications and the web to provide additional benefits and productivity. Thus, they will be motivated to adopt and use the system.

In contrast, the test results found an indirect effect of intrinsic motivation through perceived ease of use and perceived usefulness of intention to use. This is shown in Table 5 which displays the Result of Indirect Effect Test analysis. The effect of intrinsic motivation on intention to use through perceived ease of use has a t-count value of 2.84, which means > 1.64. Meanwhile, the influence of intrinsic motivation on intention to use through perceived usefulness has a t-value of 5.66, which also means > 1.64. This result shows the ease and usefulness felt by Bank Kalsel employees in using the application and the available web linking the sense of enjoyment they feel with their intention to access the application and the web as a form of receiving the digitalization process at Bank Kalsel. It is also in line with with former TAM research (Balog, 2015; Koenig-Lewis et al., 2015; Sun, 2008).

**Table 5:** Result of indirect effect test.

Path	t-value	t-table (critical value)	Path coefficient			R-square	Hypothesis conclusion
			Direct	Total Effect	Indirect		
IM → PEU → IU	2,84	1,64	0,15	0,89	0,79 ∗ 0,18 = 0,14	0,29	Accepted
IM → PU → IU	5,66	1,64	0,15	0,30	0,88 ∗ 0,54 = 0,48	0,633	Accepted

### 5.1 Implication for manager

The results of this study provide enough evidence that shows Bank Kalsel employees have a high acceptance of the use of websites and applications available in the digitalization process carried out in Bank Kalsel. In addition, Bank Kalsel employees also showed a sense of enjoyment in technology adoption through websites and applications available in their work. Furthermore, Bank Kalsel employees show acceptance in the digitalization process at Bank Kalsel has the ease of use and usefulness benefits for their performance. It shows that the employees of Bank Kalsel have positive acceptance with digitalization.

This study also recommends that Bank Kalsel's policies in this regard be directed to increase the value obtained from digitalization in completing work tasks, for example by increasing accessibility, security, and economic benefits obtained from digitalization policies undertaken. In addition, the Bank Kalsel needs to make alternatives aimed at facilitating employees and promoting a work environment that is easily adaptable to change.

The results of this study can be used as a recommendation by the company's management regarding the influence of intrinsic motivation, perceived ease of use, perceived usefulness towards the intention to use a technology system in the company. This can influence companies in the industrial sector by providing encouragement or inspiration to focus on performance, fostering the belief that using information technology will reduce certain workloads (not making excessive effort), assisting users in completing work more efficiently, and influencing the company. to generate interest in adopting a system and using new technology. In the end, it will show changes in the value of the performance received and the incentives earned.

### 5.2 Implication for researcher

This research is directed to identify and investigate the acceptance of Bank Kalsel employees in using the application and the web as a means of completing tasks and assessing performance in the digitalization process at the Bank Kalsel. This shows that all study steps are adjusted to the context of the Bank Kalsel and cannot be equated with systems and research in other contexts. Furthermore, future research can investigate other factors that become external variables in the construction of TAM. In addition, longitudinal research designs might provide better conclusions in TAM construction.

## 6 Conclusion

This study attempts to get an understanding of how employees are prepared for digitalization policies implemented in Bank Kalsel. In the end it can be seen that the staff of Bank Kalsel staff have high intention to use digitalization conducted at Bank Kalsel. Because through the intention to use employees of Bank Kalsel can foster interest in continuing to participate or take part in a particular system. It can also test employees in using applications and websites to complete their work tasks. Intention to use in this study also leads to seeing the intensity of employees in viewing and observing the results of their performance appraisals, reports on work progress, and daily information developments available in the application and website of Bank Kalsel. Indirectly, digitalization can simplify a business process and measure the performance of employees of Bank Kalsel staff. Through the implementation of digitalization, it will change the work patterns and procedures of Bank Kalsel employees from what they usually do. In addition, through the digitalization of the Bank Kalsel, it is considered that it will provide significant efficiency. This can happen because when there is a change in work processes to accommodate an increase in efficiency, the resistance to change will decrease. Trying something new becomes a trigger for enthusiasm.

In addition, this study also tries to contribute to the improvement of TAM construction. This is done by adding additional roles to the two main variables of TAM as a mediator. The finding of this additional role is expected to be a foothold in the development of TAM construction in the future.

### 6.1 Limitation of the study

This study was designed in accordance with the experience of employees in using various applications in the digitalization process in the South Kalimantan Bank, thus aspects related to the social and economic impacts of digitalization were not the focus of this study. Analysis of aspects other than the use also needs to be carried out by further research. It is important to investigate other areas that go along with the application usage process.

Furthermore, this study recommends further research to investigate the comparability of acceptance from a generational and position perspective to provide a broader understanding of technology adoption.

## Declaration

### Author contribution statement

A. Bastari: Performed the experiments; Wrote the paper.

A. Eliyana: Conceived and designed the experiments; Wrote the paper.

A. Syabarrudin: Performed the experiments.

Z. Arief: Contributed reagents, materials, analysis tools or data.

A. Permana Emur: Analyzed and interpreted the data.

### Funding statement

This research did not receive any specific grant from funding agencies in the public, commercial, or not-for-profit sectors.

### Data availability statement

Data will be made available on request.

### Declaration of interests statement

The authors declare no conflict of interest.

### Additional information

No additional information is available for this paper.
